# Hetastarch induced volume changes of starving adenoma and suggested mechanism of action

**DOI:** 10.1038/s41598-022-21693-4

**Published:** 2022-10-26

**Authors:** P. Jakabčin, M. Kello, J. Záň, J. Kolář, Jozef Uličný

**Affiliations:** 1 Department of Biophysics, Institute of Physics, Faculty of Science, Pavol Jozef Šafárik University in Košice, Jesenná 5, 040 01 Košice, Slovakia; 2 Department of Social and Clinical Pharmacy, Faculty of Pharmacy in Hradec Králové, Charles University, Prague, Czech Republic; 3 Department of Pharmacology, Faculty of Medicine, Pavol Jozef Šafárik University in Košice, Košice, Slovakia; 4 Department of Gastroenterology, Central Military Hospital-Faculty Hospital, Ružomberok, Slovakia

**Keywords:** Gastrointestinal cancer, Systems biology

## Abstract

Submucosal injection is often required step during endoscopic mucosal resection (EMR). In clinical practice we have observed that the EMR injection solution containing hetastarch (HES) lead to selective increase of the neoplasms volume, facilitating their resection. The aim of this study was to explore the possible mechanisms of such behaviour, which was not reported elsewhere. The HCT116 cell line of human colon cancer was exposed to the same EMR solution in vitro. The significant volume increase of HCT116 cells was observed, but only for starving cell culture, suggesting that the starving is essential for the neoplasms-specific volume change. We suggest, that for the iso-oncotic composition of the EMR submucosa injection solution the HES component is crucial, as it can be subject of the starch hydrolysis followed by facilitated transport of resulting monosaccharides from the submucosa into the neoplastic tissue.

## Introduction

Neoplasms of the GI tract are tumour-transformed tissues that can gradually progress into malignant tumours. The therapy of such neoplasms is usually radical and involves surgical procedure incision of detected neoplasms. The position, shape, and size of the neoplasm are typically corroborated using the endoscopic method. The endoscopist uses an endoscopic optical probe to localize the position of such neoplasms along the GI tract.

The neoplasm that is not embedded into the deeper layers of the tissue can be removed during the same endoscopic examination session by using endoscopic mucosal resection (EMR). By resection through the middle or deeper part of the submucosal layer, EMR allows complete and curative resection of the diseased mucosa. For the indicated early stages of progression, EMR can be accomplished with minimal cost, morbidity, and mortality and can improve the long-term quality of life of patient^1,2^.

Injection of a suitable solution is used to separate the neoplasm from the *muscularis propria*. If the lesion is distinct visually, it usually means that there is no deep submucosal invasion. On the other hand, the “non-lifting sign” has been found to have 100*%* sensitivity, 99% specificity, and 83% positive predictive value for invasive carcinoma^3^.

Successful elevation of neoplasms allows the application of polypectomic loop and control of the incision process. The composition of the injection solution facilitating such an elevation for EMR use is not standardized^1^, but efforts have been made to improve the coagulant and colouring properties of such compositions. In the literature, an off-label use of components approved for intravenous use is often mentioned. For example, a highly concentrated salt solution as a coagulant with the addition of epinephrine has been reported^4^. Alternatively, isotonic saline solution with the addition of derivatives of cellulose, succinyl gelatine, glycerol, and fibrinogen has been formulated to slow diffusion^1^. The physiological solution with the addition of methylene blue or sodium salt of indigotindisulfonate was applied as a visual tool to stain the neoplasm^4^. Only very recently, the ready-made injection solutions Eleview and EverLift were introduced on some markets^5^.

The disadvantage of commonly utilised off-label solutions for diagnostics and surgical treatment of neoplasms of the GI tract is mainly the short time of the elevation of the lesion (separation of adenoma) and only moderate visual distinction from surrounding tissue. The lifetime of such raised adenoma is determined by fast diffusion of injected solution, which leads to the disappearance of adenoma without its colour distinction. When an auxiliary colouring agent is used, the colour boundary between the adenoma and healthy tissue is dispersed due to the rapid diffusion of the colouring agent into both volumes.

During our search for the optimal solution composition, we found a very pronounced effect when a specific injection solution was applied as a submucosal injection into the neighbourhood of suspected neoplastic tissue as a part of the EMR procedure. Several polyps were raised above the injected tissue, lasting several minutes, allowing for comfortable application of polypectomic loop. The colouring compound formed three differently coloured volumes—injected tissue, neoplastic tissue, and the thin boundary between them, further helping to diagnose the extent of neoplastic tissue and its level of embeddedness. The new empirically found composition clearly and repeatedly improved the EMR procedure compared with the actual clinical practice at the hospital and compared with the literature.

In our attempt to elucidate the behaviour observed during EMR, we performed in vitro experiments on the HCT116 (human colorectal carcinoma) cell line subjected to the conditions present during endoscopy. In this paper, we report our observations and suggest the mechanism of action based on our limited experiments and known facts.

## Materials and methods

### Preparation of EMR solution

The EMR solution we used consists of three components: the physiological (saline) solution, in which the visual contrasting aid—the sodium salt of [4-(alpha-(4-diethylaminophenyl)-5-hydroxy-2,4disulfophenyl-methylidene)-2,5cyclohexadiene-1-ylidene]diethyl-ammonium hydroxide inner salt (Patent Blue V, solution for injection 50 mg/2 ml, GUERBET, France)^6^ and the colloid modulator of velocity –HES, (also known as hydroxyethyl starch)(VOLUVEN solution for infusion, 1 × 500 ml, Fresenius Kabi, Bad Homburg, Germany)^7^ was dissolved. We prepared a mixture of EMR composition by mixing 500 ml of isotonic saline solution with 1 ml of colour constituent (Patent Blue V) followed by diluting with a HES drawn up into the syringe with Combi-Stopper (syringe bung) in the ratio 3:7 under aseptic conditions and apyrogenic (dilution closed path) in a laminar flow hood.

### The selection of suitable patients and administration protocol

From March 1st to June 30th, 2014, 62 patients (19 females and 43 males of average age 56.8 and 61.1 years, respectively) were indicated for EMR at the Department of Gastroenterology of the Central Military Hospital. The patients were selected for EMR either directly at the Gastroenterology Department or following doctor referral at another department of gastroenterology in Slovakia due to a preventive examination. The patients were diagnosed during routine colonoscopy with sessile or semi sessile adenoma of the colon and rectum (diagnosis group ICD-10 codes D 12.0, D12.2-D12.8) with adenomas of size ranking from 5 to over 40 mm in its largest dimension. Exclusion criteria for polypectomy (EMR contraindication) were thrombocytes (PLT) less than 50 × 10^9^/l, prothrombin time ratio (INR) more than 1.4, discontinuation of anticoagulant or dual antiaggregant therapy less than seven days before EMR. The number of patients (sample size n = 62) who underwent polypectomy with EMR intervention was typical for a 4-month period at the hospital. There were no other selection and/or exclusion criteria applied. All patients within the period which qualified for EMR were administered the new EMR composition.

Informed consent was obtained from all patients. The procedure was approved by the Ethical Committee of the Central Military Hospital SNP Ružomberok, Slovakia, according to valid Slovak law^8^.

In all cases, the submucosal injection solution was prepared ahead in 10 ml syringes, containing either isotonic saline, saline with epinephrine or saline with Methylene Blue or Patent Blue V. The new EMR composition was prepared in the same 10 ml syringe form with composition as specified above.

The administration protocol followed the routinely used procedure and was performed in accordance with the relevant guidelines and regulations. The only change in the standard application protocol was replacing the syringes containing a physiological (saline) solution, Methylene Blue/Patent Blue V, and epinephrine with the syringes containing new composition.

During the endoscopic session, the close neighbourhood of suspected tissues was injected by submucosal injection via the endoscopic channel leading to bolus formation in submucosa and subsequent volume and colour changes in the area. Depending on the size of initially observed polyp, the amount of injected EMR solution was 1–3 ml for small, 5 mm diameter polyps, the larger lesions e.g., 40 × 50 mm surface required 50–80 ml of EMR solution. There were no noticeable differences in volumes of EMR solution compared to the typical volumes of EMR solutions applied before. When new polyps were elevated and became visible in the field of view, the additional EMR solution was applied, when necessary. The elevated polyps were removed using a polypectomic loop, and removed tissue was analysed for histology. As a rule, several polyps became visible lasting several minutes and were removed in a single step during the same endoscopic session. En-bloc resection rate was not significantly different for the new solution. Generally, polyps below 20 mm size were resected en-bloc, while above that size, piece-meal resection was more typical. In some cases, polyps of about 30 mm size could be removed en-bloc.

### Cell line model

The human colorectal carcinoma cell line HCT116 was purchased from American Type Culture Collection (ATCC, CCL-247) and cultured in RPMI 1640 growth medium (Biosera, Kansas City, MO, United States). The growth medium was supplemented with 10% foetal bovine serum (FBS) and 1× HyClone Antibiotic/Antimycotic solution (GE Healthcare, Little Chalfont, UK) and maintained in an atmosphere containing 5% CO_2_ in humidified air at 37 °C. Before experiments, the viability of cells was analyzed by trypan blue assay. The cell line was authenticated by the ATCC Laboratory Authentication Service using Sanger sequencing. ATCC declared no Mycoplasma contamination. Before experiments, cell specimens were tested again for Mycoplasma contamination by DNA staining and fluorescence microscopy visualization with negative results.

For experiments, cells were seeded in 96-well low-density plates and maintained in complete culture medium for 24 h. The cells were then starved in saline solution without nutrients for 24 h, mimicking the patient preparation before EMR surgery.

The initial cell culture was grown to 10 thousand cells per well, forming plaques. During starvation, some of the cells detached and floated freely in the medium. We removed free-floating cells with the part of liquid media, and the removed volume was then restored by adding the same volume of saline solution. Even though the saline solution detaches part of the starving cells, a significant fraction of the starving cells remains attached to the density plate after adding the saline solution.

### Adding the contrasting composition

The EMR composition was applied for two groups of cells—nonstarving and after 24 h starving in saline solution. After 24 h, the nonstarving cells adhered to the density plate, while in starving cells, the free-floating cells had to be removed, and the media was replenished by saline solution first. In both cases, the last step of the procedure consisted of replacing the saline medium with an EMR composition. For starving cells, approximately half of the volume of saline was replaced by EMR. The estimated volume of the saline solution replaced by EMR composition was in 100 µl range. Subsequently, we prepared a set of substances without a colour constituent. In all cases, the cells exposed to the solution were followed by 10 min of live video flow on a Cytation 3 Cell Imaging multimode sensor (BioTek Instruments, Inc.) and evaluated visually for cell count, cell volume, and cell shape change.

## Results

### EMR use

**Figure 1:**
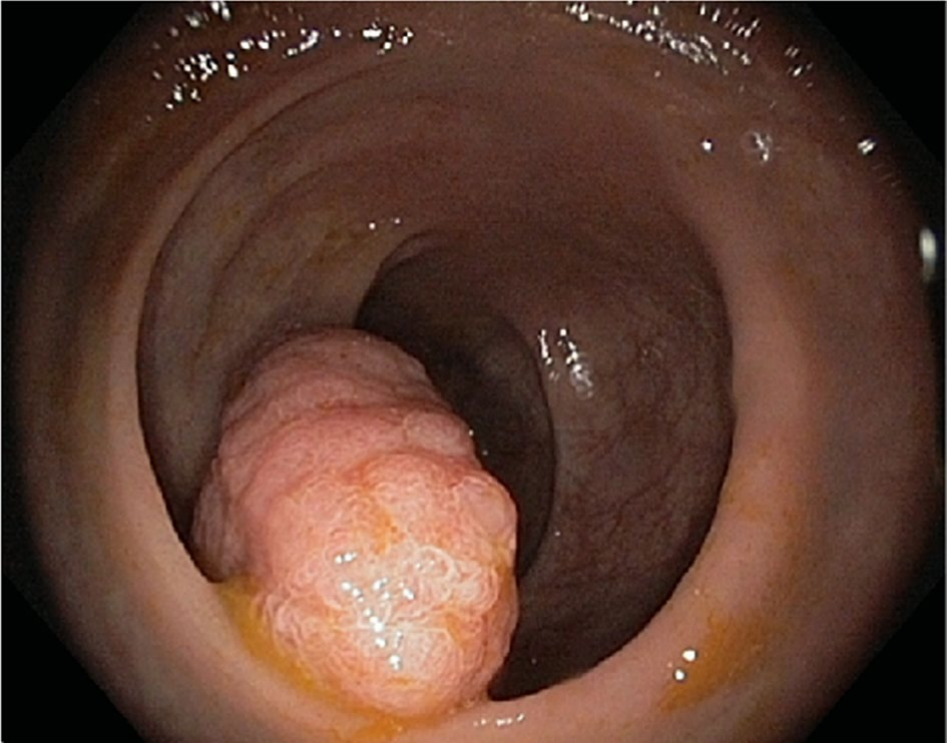
Endoscopic image. The adenomatous polyp immediately before submucosal injection of the EMR composition. The larger dimension of the polyp is about 45 mm.

**Figure 2:**
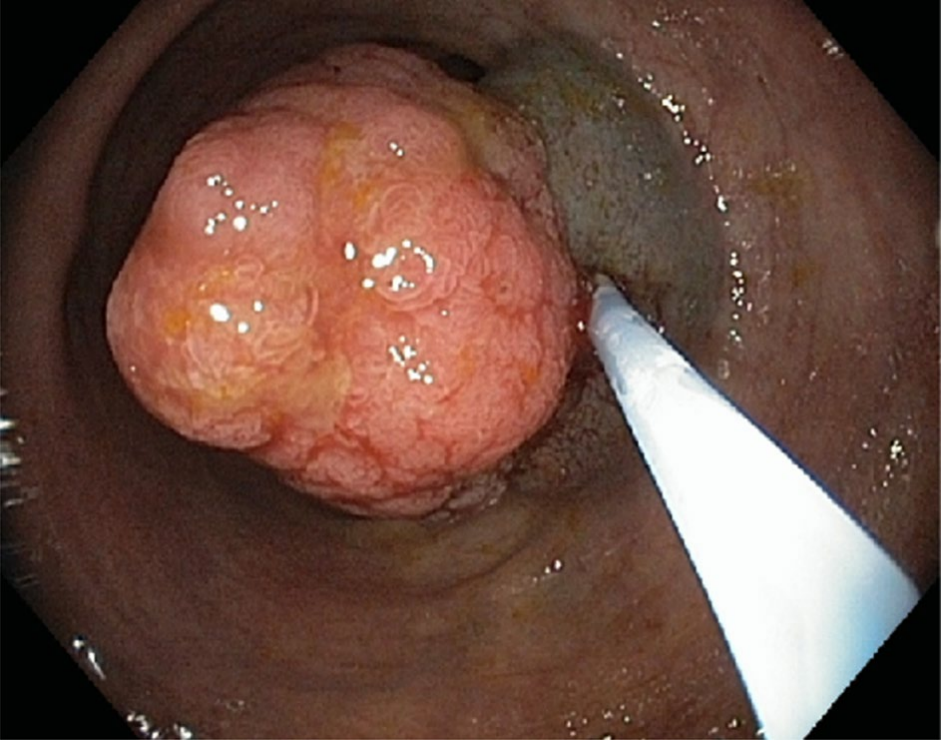
Endoscopic image. The same polyp as in Fig. 1. Submucosal injection of EMR composition below the adenoma. Image taken 150 s after the submucosal injection.

**Figure 3:**
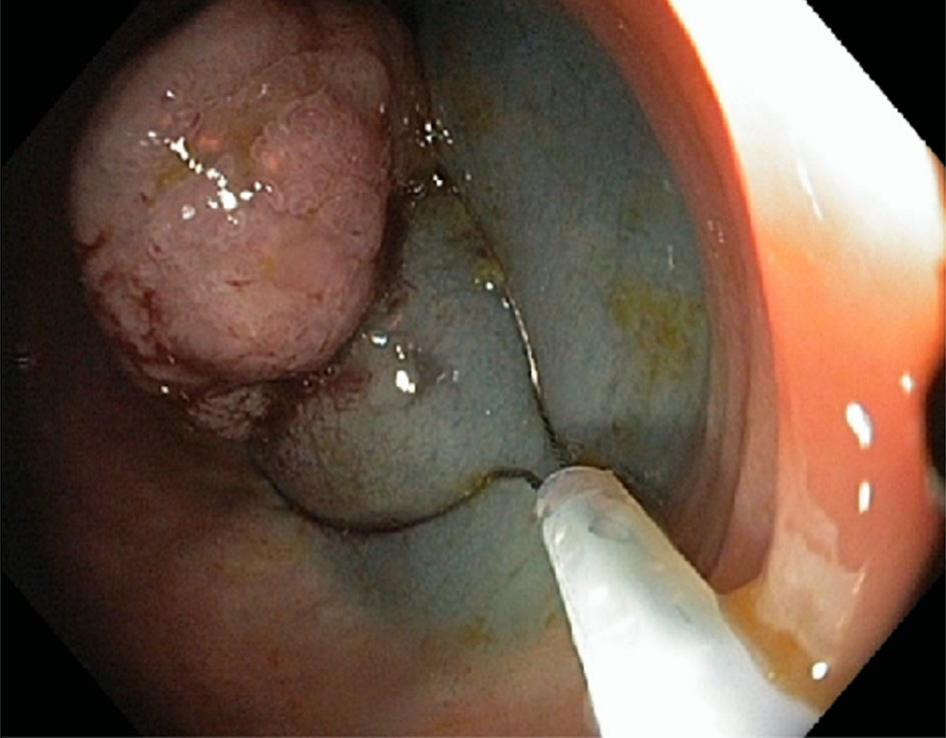
Endoscopic image. The same polyp as in Figs. 1 and 2. The polypectomic loop is inserted 300 s after the submucosal injection of EMR composition.

**Figure 4:**
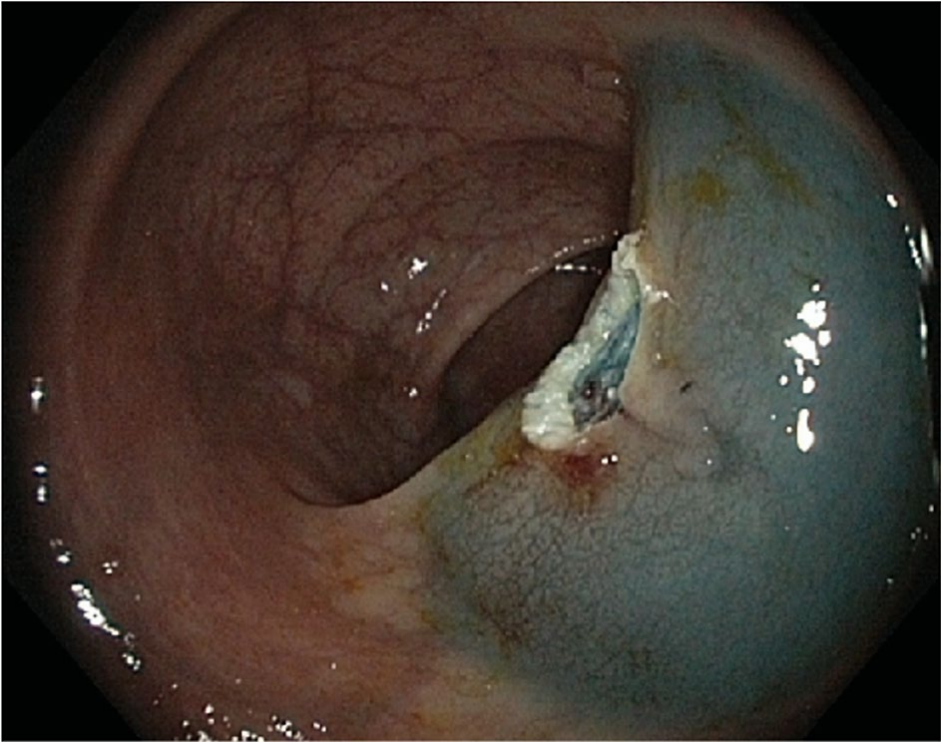
Endoscopic image. En-bloc resection and complete removal of the GI adenoma depicted in Figs. 1, 2 and 3. 330 s after the submucosal injection.

Under the conditions specified above, the EMR composition was applied to all 62 patients with adenoma qualifying for EMR within the period March–June 2014. In the absence of standardized EMR solutions, such as Eleview or EverLift (both appeared only later on the market and are still not registered for use in Slovakia), we could compare only the effects against the same EMR procedure, as performed outside the experimental period with commonly used off-prescription solutions. The differences against saline solution commonly used at the hospital were striking. The initial bolus in submucosa gradually diminished in size while the surrounding polyps started to elevate, rise their volume, and change the colour at the boundary. The typical reaction to the administration of contrasting composition by submucosal injection of adenoma larger than approximately 20 mm is depicted in Figs. 1, 2, 3 and 4. EMR composition provides colour-contrasting differences between the tissues. The normal tissue is light blue. The thin boundary of dark blue colour is formed between the adenoma and the healthy tissue, while the adenoma itself is not coloured. By injecting this EMR composition into the submucosal layer, adenomatous polyps noticeably increase their volume and elevate above the surface for 10–25 min, prolonging the time window for resection. At the same time, sharp colour differences between the healthy and neoplastic tissues and the boundary between them can be observed. Colour distinction and the increased volume of the elevated polyp thus improved the precision and quality of polypectomic surgery. The observed reaction of adenomatous polyps to new EMR composition differs from reaction to commonly used solutions where the volume changes are in the submucosa and the colour contrast—if colouring component is used—is more diffuse and not so sharply pronounced. Even in comparison with the recently introduced Eleview and EverLift contrasting solution we could note two significant differences. (1) The reported elevation time of Eleview is longer (up to 1 h) compared to slightly over 15 min in our observation. (2) more importantly, the described behaviour of the polyps after the submucosal injection of both Eleview and EverLift lack (a) the elevation of the other submerged polyps in the proximity of injection and (b) volume changes of the polyp itself. Interestingly in the paper comparing the Eleview vs HES contrasting composition authors did not notice the volume change of polyps themselves, focusing rather on bolus volume dynamics^5^. While the elevation time of Eleview is excellent, the differences in volume changes lead us to believe, that the EMR solution activity based on HES is on first sight similar but acts on different mechanism. This seems to be plausible difference of dynamics based on different chemical composition.

### In vitro model

**Figure 5:**
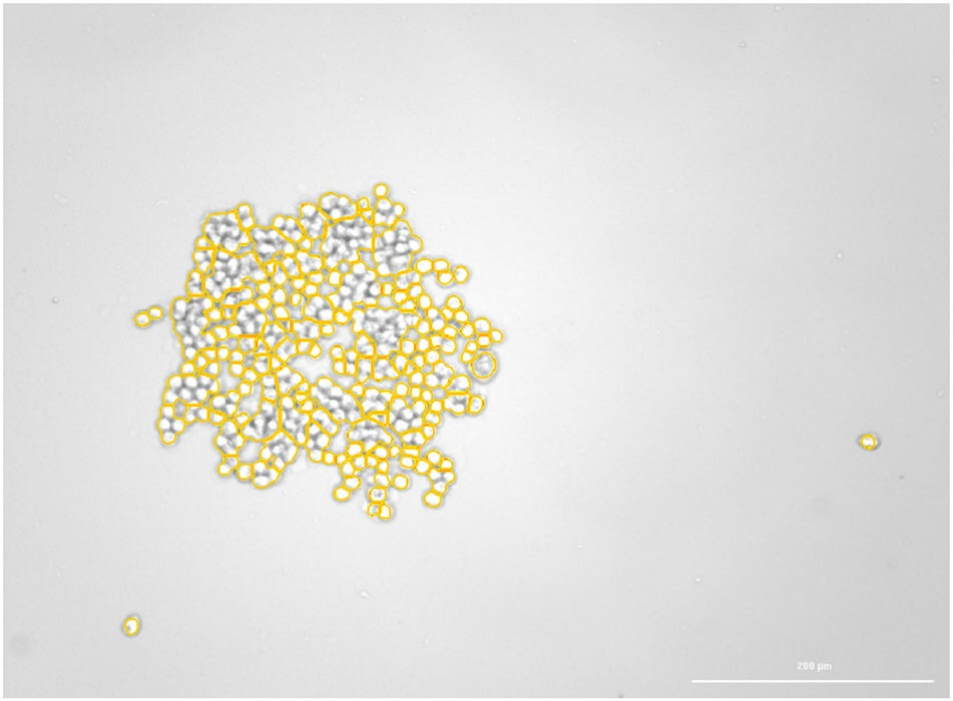
Microscopic image. Group of starving HCT116 cells with free-floating cells removed (bright-field image taken by Cytation 3 Cell Imaging multimode sensor). The scale bar on bottom right of the picture corresponds to 200 µm.

**Figure 6:**
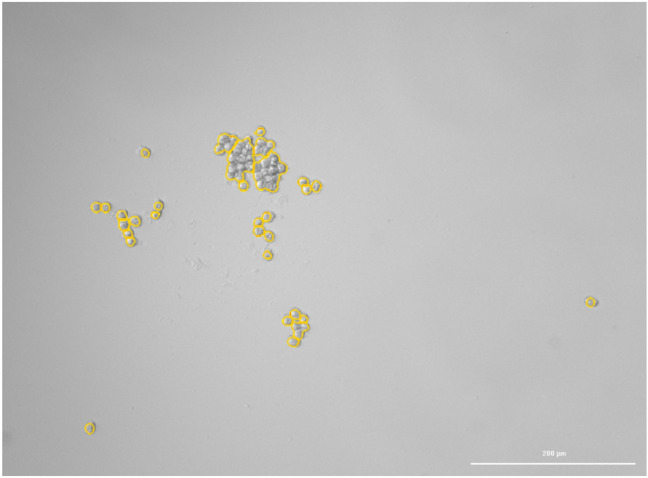
Microscopic image. The same field of view as in Fig. 5 after the cells were exposed to combination saline solution and HES at a ratio of 3:7. After T + 30 s, the cells detach and, for most part, float away from the field of view (bright field image taken by Cytation 3 Cell Imaging multimode sensor). The scale bar on bottom right of the picture corresponds to 200 µm.

For nonstarving cells, we observed no significant changes when the saline solution was replaced by EMR solution. This was expected for iso-oncotic injection solution. This is in sharp contrast with the reaction of starving HCT116 cells. In repeated experiments, HCT116 cells starved for 24 h in saline solution detached entirely from the density plate. Figures 5 and 6 show rare cases where the group of detached cells remained partially attached to the bottom of the well-plate so that the cells could still be localized and recognized. In other cases, the expansion of cells was so pronounced that the cells completely disappeared from the visual field of the microscope and were not identified. For the cells still attached in both Figs. 5 and 6, we estimated cell volume rises of approximately 4%. Nevertheless, for most experiments, the expected volume expansion leading to the separation of cells must be higher but was not quantified.

The experiments repeated after two weeks with different batch of HCT116 cultures confirmed the same results.

## Discussion

Limitation of the study. The clinical observation was made during routine run of the Department of the Gastroenterology. The number of patients were typical for the period and size of the hospital. No blind study was performed – all patients were subjected to uniform treatment—the same EMR composition containing HES, prepared from stock solution, so that the comparison can be made only with the patients subjected to EMR at the same hospital at different time. Quantification of the volume changes in complex endoscopic environment was not done.

Despite this, the clinical observation made using HES addition to commonly used EMR solutions yielded clear improvement of the EMR practice.

The complexity of the adenomas or carcinomas of the colon is well known and the cautions about the generalisation of the simple in vitro models are justified. The complex tissue is composed of several cell types in healthy architecture disturbed by neoplastic tissues in various stages of development.

Still, the HCT116 cell line should be good representative of the major constituting cell component of adenoma and/or carcinoma, responsible for the volume change effect.

The only new component in the composition is HES and the only mechanism to cause different behaviour we could think of is based on HES hydrolysis and subsequent rapid transport of resulting monosaccharides into the neoplastic cells’ interior. We found no direct experiments of others to point to the early changes in starch processing enzymes involved in adenoma-carcinoma sequence, but also, we found no data/publications directly contradicting such development. Clearly, additional experiments are much needed to further elaborate or to disprove the suggested mechanism. In colorectal carcinoma^9^—at the end of the adenoma-carcinoma transition sequence, the anaerobic glycolysis requires enormous consumption of glucose (monosaccharides) and newly acquired starch processing capabilities of neoplastic tissue might be an important step. The question of when and how the transition from normal to cancerous cells occurs is not sufficiently researched.

Our original attempt to improve the EMR composition led us to observe significant differences in the response exclusively for starving cells. Starvation of in vitro cell culture was achieved by leaving the cells 24 h in saline only (i.e., no standard fasting medium). In clinical practice, starvation of the cells is accomplished by the patient abstaining from oral food and fluid intake for 24 h (*nill per os*) before EMR.

The administration of EMR composition into submucosa exposes the embedded part of the adenomatous polyp to HES-chemically modified starch. HES, as a large macromolecule (average molecular weight 130 kDa)^10^, is expected to diffuse slowly in the submucosa. Indeed, this can be seen by comparison of Figs. 2 and 3 separated in time by 150 s.

We expect to find alpha-amylases (or functionally equivalent enzymes) present in the submucosa and released to the environment by starving neoplastic cells. While we found no direct data supporting the expression of starch-processing alpha-amylases in colon adenomas, they are reported in similar and thus related lung adenocarcinomas^11^. Under the conditions indicated for EMR, adenoma polyps of size approximately 20 mm are still mostly benign—less than 10% further progress along a known adenoma-carcinoma sequence^12^. The probability of 62 EMR resections being all carcinoma is thus very low, and we shall assume that adenomas already express alpha-amylases in sufficient amounts. Due to the applied protocol, we can assume that alpha-amylases are present even when part of the liquid (the saline solution used for starving) is removed; on the time scale of experiments, the de novo synthesis of alpha-amylases should not manifest within tens of seconds of HCT116 exposure to EMR.

If alpha-amylases (or their functional equivalents) are present in the submucosa, the initial volume of iso-oncotic HES can be degraded, and progressively smaller hydrolysis fragments form in the submucosa. In contrast to blood plasma, even gradual hydrolysis to fragments below the renal threshold (45–60 kDa)^13^ remains active in the submucosa. As a result, a continuous supply of glucose and hydroxyethyl glucose (hydroxy ethylated at C2/C6 ratio 9.05:1)^10^ is delivered into the submucosa. The dynamic mixture of fragments, including the final monosaccharide product of alpha-amylases, is produced in the submucosa, contributing to the rise of oncotic pressure.

Without the transport of monosaccharides, oncotic pressure manifests as a volume increase in the submucosa. In our observation, however, the dominant volume changes were observed in the volume of neoplastic tissue. A similar volume change was also observable in vitro in adherent 2D plaques formed by the model HCT116 line. While carcinomas are known for enhanced expression of glucose transporters, adenomas must gradually acquire the capability in the early stage of adenoma-carcinoma transition. It seems thus reasonable that polyps of approximately 20 mm size, indicated for EMR, already possess an enhanced amount of glucose transporters, particularly GLUT1^14,15^.

In healthy tissue, the transport of monosaccharides proceeds in the direction from the lumen to the serosa, and the transport for an excessive concentration of monosaccharides is facilitated. The adenomas expose the same surface present to the lumen to the submucosa so that the direction of facilitated transport of monosaccharides is reverted.

The facilitated transport of sugars into cells is specific because different saccharides are transported with different efficiencies. Additionally, hydroxyethyl glucoses are expected to be transported into cells less efficiently than anhydrous glucose. Thus, the depletion of the pool of oncotic pressure generating saccharides in the submucosa volume follows complex kinetics, well beyond our current focus.

Nevertheless, the in vitro HCT116 model cells eagerly transport oncotic molecules, as demonstrated by violent volume changes leading to loss of cell adherence. This kinetics can be the reason why the polyp rather than the submucosa increases the volume in clinical observation/application.

## Conclusion

On the practical side, the modification of EMR composition and the application protocol lower the application barrier for EMR by improving the comfort and precision of the EMR. By clearly delineating the polyp boundary and the volume changes, lasting a longer time, EMR can be performed with less time-related stress and a lower risk of unwanted complications.

Moreover, our work suggests—somewhat surprisingly—that starving adenomas in the early stage of transition adenoma-carcinoma already express alpha-amylases and exhibit elevated glucose transport responsible for volume changes. This—if confirmed by additional experiments—provides the opportunity for functional diagnostics similar in spirit to fluorodeoxyglucose contrasting in PET^2^.

## Data Availability

The datasets generated during the current study are not publicly available because they contain sensitive patients’ data. Archived hospital records, including patients age, sex, ATC diagnoses and other relevant pre-colonoscopy records, histology of the extracted tissues taken during the EMR as well as the number of repeated colonoscopy sessions are available after anonymization from authors at reasonable request.
